# Intradural Disc Herniation in the Lumbar Spine: A Case Report

**DOI:** 10.31729/jnma.4798

**Published:** 2020-05

**Authors:** Tushar Rathod, Sameer Panchal, Nandan Marathe, Yogendra Agrahari, Ashwin Sathe

**Affiliations:** 1 Department of Orthopedics, Seth Gordhandas Sunderdas Medical College and King Edward Memorial Hospital, Mumbai, India; 2 Department of Orthopedics, Shree Tinau International Hospital, Butwal, Nepal

**Keywords:** *disc, herniated*, *discectomy*, *intradural*, *lumbar spine*

## Abstract

Intradural disc herniation is a rare presentation of a common pathology, comprising around 0.28-0.3% of all disc herniations. It occurs when disc material related to an intervertebral disc penetrates the spinal dura and lies in an intradural extramedullary location. A 60 years old male patient presented with complaints of low back pain and right lower limb radiculopathy of 2 weeks duration. Neurological examination revealed the weakness of extensor hallucis longus and ankle dorsiflexion with diminished sensation corresponding to fourth and fifth lumbar (L_4_-L_5_) dermatome on the right side. Magnetic resonance imaging showed a large sequestered fragment with intradural extensions and posterior longitudinal ligament tear. Intradural nerve root showed significant displacement with severe central canal and right lateral recess stenosis. Discectomy was performed along with the removal of the intradural extension. The postoperative course was uneventful.

## INTRODUCTION

Rupture of intervertebral disc material into the intradural space is a rare event in lumbar disc disease but must be considered in the differential diagnosis of mass lesions causing nerve root impingement or cauda equina syndrome.^1^ The pathogenesis of lumbar intradural disc herniation is not known clearly but is most likely related to dense adhesions between the ventral dura mater and the posterior longitudinal ligament. These adhesions can result either from repeated minor trauma or from prior spine surgery.

## CASE REPORT

**Figure 1:**
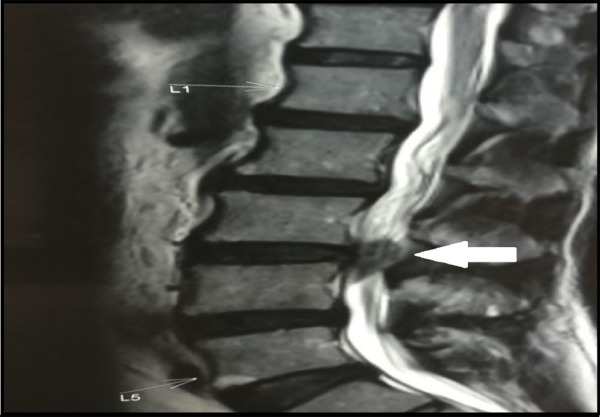
Preoperative 3 Tesla MRI lumbar spine T2 weighted sagittal section.

A previously healthy 60 years old male presented with complaints of low backache and pain radiating to his right leg for 2 weeks. He had difficulty walking with weakness in the right lower limb. He did not respond to conservative treatment. He had no history of trauma or previous history of spine surgery. Neurological examination revealed a medical research council (MRC) grade 0 power in right side ankle dorsiflexors and extensor hallucis longus (EHL). The straight leg raising test was positive on the right side at 50 degrees. Preoperative magnetic resonance imaging (MRI) revealed the presence of a large sequestered disc with intradural extension at the third and fourth lumbar vertebrae (L_3_-L_4_) level with a tear in a posterior longitudinal ligament (Figure 1).

**Figure 2:**
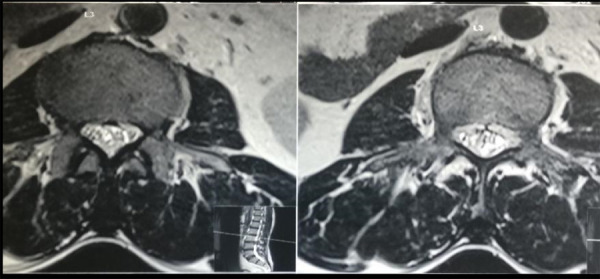
Preoperative 3 Tesla MRI LS spine T2 weighted axial section at the L_3_-L_4_ level.

There was also a significant residual disc in the central canal and right lateral recess stenosis (Figure 2). Preoperative nerve conduction study showed bilateral (right > left) L5 active plus chronic motor axon degeneration with partial conduction block at root level suggestive of recent ongoing involvement.

**Figure 3:**
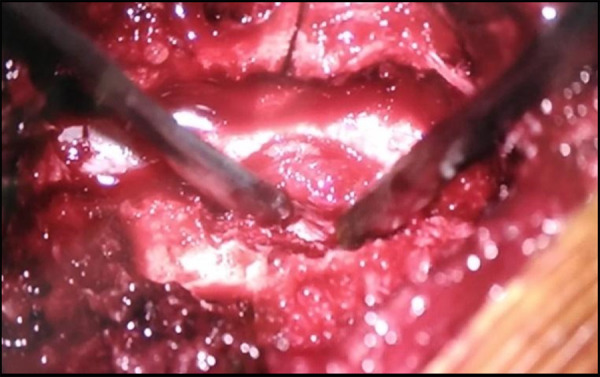
Intraoperative image of disc adhered to the dura.

Under general anesthesia and in the prone position, midline laminectomy was done at the L 3-L level with careful preservation of the facet joint capsule. After the adequate release of adhesions (Figure 3), a large sequestered disc fragment invading the thecal sac on the right side causing significant compression around the origin of the right L nerve root was noted.

Thorough discectomy was performed with meticulous removal of intradural extension which led to the opening in thecal sac and dural leak. Dural tear repair was performed with a prolene 5-0 suture. Appropriate closure was confirmed on the table by the Valsalva manoeuvre to check for leakage of cerebrospinal fluid.

**Figure 4:**
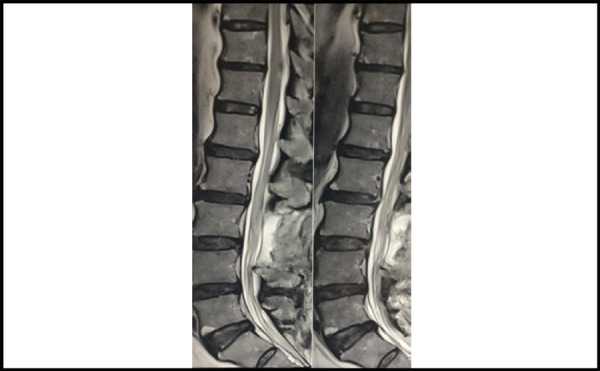
Postoperative 3 Tesla MRI LS spine T2 weighted sagittal view.

**Figure 5:**
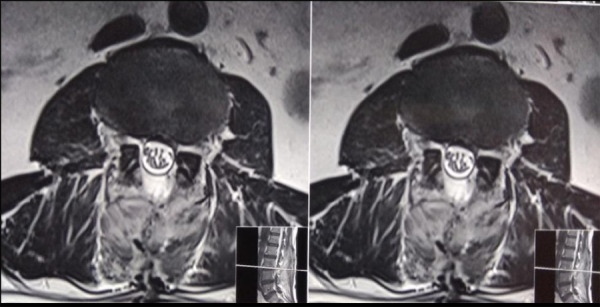
Postoperative 3 Tesla MRI LS spine T2 weighted axial section at the L3-L4 level.

Post-operative MRI was done 2 weeks post-surgery which showed postoperative changes in the form of laminectomy defects. Postoperative discectomy changes were seen at L-L level with complete _34_ restoration of thecal sac without any compromise in the spinal canal (Figure 4 and Figure 5).

The patient had an uneventful postoperative course with complete resolution of right lower limb radiculopathy. We also noted neurological recovery in the form of improvement in right-sided ankle dorsiflexion and EHL power to grade 3 (MRC) from grade 0 (MRC). At present, the patient has grade 3 (MRC) power and no other complaints at a 2-year follow-up.

## DISCUSSION

Intradural disc herniation is a rare but important cause of radiculopathy and cauda equina syndrome. As a comparatively rare pathology, the first report of an intradural disc herniation was presented by Walter E. Dandy^1^ in 1942. Approximately 123 cases of IDH have been reported since 1942.^2^The majority of the intradural herniations have occurred at the L-L levels with 12 cases reported at the L-S_1_ level. The most frequently affected site is L-L(55%), followed by L-L (16%) as seen in this case and L-S_1_ (10%).^3^ A classification system has been proposed for IDH: Type A: Herniation of a disc into the dural sac; Type B: Herniation of disc into the dural sheath in the preganglionic region of the nerve root.^4^

It is not easy to explain how the nucleus pulposus perforates the dura and why this phenomenon occurs most frequently at the L -L level.^4^ Some histology and anatomy textbooks state that the narrow epidural space contains loose connective and fatty tissue and venous plexus and that the spinal dura mater is attached to the circumference of the foramen magnum and to the posterior surfaces of the bodies of second and third cervical vertebrae; it is also connected by fibrous slips to the posterior longitudinal ligament, especially near the lower end of vertebral canal.^5^,^6^

The explanation of the dural perforation by the disc herniation is not clear though several reasons are known that may contribute like congenital narrowing of the spinal canal with less epidural space, adhesions between the annulus fibrosus, posterior longitudinal ligament, dura mater, congenital and iatrogenic fineness of the dura mater.^5-7^ It is postulated that dense post-traumatic or postoperative adhesions between the posterior longitudinal ligament and the ventral dura prevent the lateral migration of disc fragments and facilitate penetration of the tethered dura. An anatomical investigation revealed dense nonseparable adhesions of the ventral dura to the posterior longitudinal ligament at the L -L level in eight of 40 cadavers. It was suggested that adhesions formed congenially or caused by trauma, surgery, inflammation, osteophytes, or disc protrusion fixed the dural sac. In those cases, the extruded fragment tore the ventral surface of the dura. Blikra demonstrated the presence of firm anatomic adhesions between the anterior wall of the dural sac and the posterior longitudinal ligament, particularly at the L -L level.^8^ This theory only explains the intradural disc herniations at the L -L level.^8^ Multiple imaging modalities have been described to detect IDH. Imaging IDH accurately requires a contrast-enhanced MRI. However, unfortunately, this is not routinely advised in patients presenting with back pain and radiculopathy. Contrast-enhanced MRI is crucial for both diagnosis and to differentiate a herniated disc from disc space infections or tumor.^9^ Penning and Wilmink showed in their flexion-extension myelographic study that the dural sac was strained to various degrees at different levels.^10^ The most extreme strain occurs at L-L levels which can lead to the weakening of dura at the levels in question.

A herniated disc fragment will rarely be enhanced centrally,which is attributed to vascular granulation tissue infiltrating the fragment.^9^ Hodge and Binet demonstrated that intradural disc rupture can be diagnosed pre-operatively by utilizing metrizamide enhancement of spinal computerized tomography.^11^ Existence of macrophages in the cerebrospinal fluid obtained at myelography may also suggest the diagnosis.^12^

The intradural finding of intervertebral disc material is a rare phenomenon. The reason why the number of reported intradural disc herniation is quite low may be that these cases are not documented or recognized. Biomechanical studies, in addition to anatomical studies, may improve our understanding of pathophysiological processes in these patients. While treating lumbar disc disease, the possibility of an intradural disc herniation should be kept in mind for the success of the discectomy and the management of the failed back syndrome. The diagnosis is commonly an intraoperative surprise because contrast MRI is not routinely advised for radiculopathy.

**Consent:** JNMA Case Report Consent Form was signed by the patient and the original article is attached with the patient’s chart.

## Conflicts of Interest:

**None.**
